# Transition Between Sensitive Delusion of Reference and Mood Disorder: A Case Report

**DOI:** 10.3389/fpsyt.2021.712552

**Published:** 2021-09-06

**Authors:** Cecilia Maria Esposito, Alessio Fiorentini, Antonio Callari, Gian Maria Galeazzi, Paolo Brambilla

**Affiliations:** ^1^Department of Neurosciences and Mental Health, Fondazione IRCCS Ca' Granda, Ospedale Maggiore Policlinico, Milan, Italy; ^2^Department of Biomedical, Metabolic and Neural Sciences, University of Modena and Reggio Emilia, Modena, Italy; ^3^Dipartimento di Salute Mentale e Dipendenze Patologiche, USL-IRCCS di Reggio Emilia, Reggio Emilia, Italy; ^4^Department of Pathophysiology and Transplantation, University of Milan, Milan, Italy

**Keywords:** mood disorder, sensitive delusion of reference, psychopathology, bipolar disorder, kretschmer

## Abstract

The Sensitive Delusion of Reference is a clinical entity described by Ernst Kretschmer and never integrated into mainstream nosographic systems. It represents the possibility of developing psychosis starting from a personality characterized by sensitivity, scrupulousness, and fear of judgment of others. The presentation of the following clinical case highlights how the overlap between this clinical entity and mood disorders leads to characteristic psychopathology, which has not been sufficiently detailed. In particular, the delusions, which always starts from the idea of reference and the shame in the face of the judgment of others, takes on characteristics of guilt during the depressive phases and persecutory themes during the activation phases. This clinical observation, which obviously needs to be confirmed on a larger scale, encourages a renewed interest in the concept of Kretschmer's Sensitive Delusion of Reference and creates the possibility of intersecting multiple psychopathological levels, for a more complete perspective on the individual case.

## Introduction

The Sensitive Delusion of Reference is a clinical entity described by Ernst Kretschmer in 1918 and never integrated into mainstream nosographic systems. It represents a paradigm of the continuity between personality and delusional outbreak within the historical debate between those who conceive psychosis to be a process or a development in Jaspersian terms (1–3). In fact, those who understand psychosis as a process underline its discontinuity with the person's temperament and previous life events. They argue that psychosis is an organic morbid entity that at a certain point in life creates a gap, which makes psychotic experiences incomprehensible to the observer. On the contrary, understanding psychosis as a development means appreciating its continuity with the personality prior to the onset of the disorder, and understanding delusions as the end result of a vulnerable constitution, and as an entity understandable in the light of the life history of the patient (4, 5). Kretschmer, in describing these particular types of paranoid delusions, offers a prototype of this second position: the Sensitive Delusion of Reference is presented as the understandable development of psychotic symptoms starting from a personality with specific characteristics, triggered by a key event and often occurring in a particular social milieu (1).

Current nosography, in particular, the most recent version of the Diagnostic and Statistical Manual of Mental Disorders (DSM-5) (6), does not allow the classification of the clinical picture described by Kretschmer within a single category, making it necessary to list various comorbid diagnoses, as will be seen in the case report below. Other nosographic systems would allow diagnoses that are more similar to those described by Kretschmer. For example, the diagnosis of Psychogenic Paranoid Psychosis (code: F23) is described in the International Classification of Disease, Tenth Revision (ICD-10) (7). In fact, in this case, although there is no reflection on the personality characteristics that would make the development of psychotic reactions easier, the psychogenic and reactive origin of psychotic episodes is nevertheless conceived. Although, the RdoC project does not produce an autonomous and alternative nosographic system in respect to the classifications already discussed, it nevertheless represents an important opportunity for the integration, which is fundamental in the study of complex cases (8).

The sensitive personality described by Kretschmer is characterized by the tendency to experience social withdrawal on a phobic and avoidant basis, by scrupulousness and conscientiousness, and by a marked sensitivity to interpersonal judgment (1, 9). The sense of inadequacy and shame is typical of the social interactions of sensitive characters that persist throughout their lives, often leading to a dysthymic temperament and the onset of anxiety disorders of a predominantly obsessive matrix. However, In the presence of particular environmental triggers, the shame from the judgment of others can take on the characteristics of a persecutory delusion (10, 11). The causes of psychotic symptoms can be hyperacute, chronic, or relapsing-remitting. According to Kretschmer, these delusional states have a benign prognosis (1).

The effective oscillations in the clinical cases described by Kretschmer were mainly represented by depressive episodes focused on themes of guilt. Manic episodes are not described; indeed particular attention is paid to the differential diagnosis with Manic-Depressive Illness, currently known as Bipolar Disorder. In particular, the elements that would make it possible to differentiate the two clinical entities would be the cyclicality present in Bipolar Disorder and absent in the Sensitive Delusion of Reference, as well as the triggering effect of the environmental factors on the onset of the episodes and the core presentation with delusions in the case of the Sensitive Delusions of Reference. Kretschmer, however, did not exclude that there may be overlaps and comorbidities between these two clinical entities (1).

The clinical case which came to our observation depicts a long history of illness showing the relationship between Sensitive Delusion of Reference and Affective Disorders. Our patient, who undoubtedly presents a sensitive personality, also has affective swings in his clinical history that typically hinges on his personality development.

## Case Report

C.M. is a 56-year-old man followed as an outpatient in mental health services for more than 20 years. Since adolescence, he was described as a shy, reserved person, and extremely sensitive to interpersonal interactions. He has always found himself at ease only with his family, having few friendships, however short-lived, and no significant romantic relationship. He reports that he has always found himself oscillating between the search of fulfilling a social relationship and his fear because he has always felt inadequate, and at fault when interacting with others. He always had a good academic performance, until he graduated in law.

The onset of the illness took place at the end of university when C.M. experienced a depressive episode. The environmental trigger seemed to have been a failed university course exam, after which the patient reported that he gradually developed the feeling that everyone was aware of his failure, and that they judged him for it. The extreme interpersonal sensitivity reached delusional characteristics during this period, structuring a real delusion of reference with persecutory aspects. C.M. described an experience of blame and shame, which he felt around him when he met people on the street, but also by what he saw on television or read in books. Ideas of guilt grew and worsened by the conviction that others could “read his mind” and therefore were aware of countless reasons to blame him. At that time, he reported the presence of derogatory auditory hallucinations blaming him. The episode, which lasted several months, resulted in a delay in achieving his degree, and then it resolved spontaneously and progressively without him seeing a psychiatrist. Although the episode was not accompanied by the execution of psychometric scales since the patient did not go to a psychiatrist, the characteristics of the episode satisfies the criteria for severe Major Depressive Episodes with Psychotic Characteristics, from the evaluation of the Structured Clinical Interview for DSM-5 Clinician Version (SCID-5-CV) regarding previous episodes (12). Later, he described a period of increased energy, busyness, decreased need for sleep, and increased self-esteem, during which the patient enrolled in the faculty of political science, wanting to obtain a second degree. However, he stopped after taking a few exams, due to the recurrence of depressive symptoms.

When he started treatment as an outpatient in psychiatry services, C.M., 36 years old, already had a long history of depression. He described symptomatology characterized by recurrent thoughts of guilt and inadequacy, mostly experienced in an ego-dystonic way, in which he patient complained of a sense of discomfort. There are also mental compulsions, consciously enacted in an attempt to ward off unpleasant thoughts. His symptoms are categorized as Obsessive-Compulsive Disorder, which leads to the commencement of psychopharmacological treatment. The prescription consisted of a high-dose antidepressant, sertraline up to 200 mg, and an antipsychotic, olanzapine up to 20 mg/day.

On regular basis, C.M. attends psychiatric appointments, and started attending rehabilitation activities, while retaining the tendency to socially isolate. Despite adherence to therapy, total remission of symptoms is never achieved. Over the years, his mood presents alternations between periods of depression, in which obsessive symptoms become more stubborn and disabling, and periods in which the patient feels “high,” active, less uncomfortable in social relationships, and less pervasively dominated by obsessive ideation. These activated phases never amount to real manic episodes but are characterized by increased energy, dysphoria, restlessness, and a greater drive to build social relationships. During mood episodes and intercritical periods, a marked interpersonal sensitivity with a tendency to misinterpret remains. The ideas of reference are experienced at times in an ego-dystonic way, as obsessive ideas, while at times in an ego-syntonic way, as authentic delusional ideas. In periods in which these ideas became more intense, haloperidol up to 2 mg/day is added to his prescriptions. The fluctuation of the thought content is congruent with the mood alternation, configuring prevailing experiences of inadequacy and guilt during the depressive phases and prevalent experiences of persecution during the activated phases.

C.M. spends his life in an increasingly withdrawn way, struggling to interact with people also at work, therefore a disability pension is allocated to him and he continues only to carry out accounting work at his sister's office. He lives alone, in the same building where his mother also lives, with whom he spends most of the day. He still has very few social relationships and no romantic relationships.

In March 2020, his habitual stability was disrupted by the Covid-19 pandemic and the consequent lock-down. In addition to having to stop going to work, he was left to assist his mother almost alone, who had recently been diagnosed with Alzheimer's dementia. A depressive exacerbation began, and reached its peak in August. He was then voluntarily admitted to the psychiatry ward, sent by his psychiatrist, who found him completely blocked during an outpatient visit.

Upon entering the ward, he appeared slowed down from a psychomotor point of view, gloomy, reticent, and distressed. After a few days, he reported that when admitted he felt as if he was in hell, and thought that the Day of Judgment would soon arrive. His thoughts appeared to be dominated by ruminations of guilt, experienced in an ego-syntonic way so much that C.M. appeared busy reviewing all his life looking for reasons for blame. He also reported that he took advantage of his family, but appeared confused and unable to explain how or when. Rating scales administered at admission show severe mental impairment: Brief Psychiatric Rating Scale (BPRS) = 69 (13), Hamilton Rating Scale for Depression (HAM-D) = 22 (14), Hamilton Anxiety Scale (HAM-A) = 19 (15), Young Mania Rating Scale (YMRS) = 9 (16), Positive And Negative Symptoms Scale (PANSS) = 87 (17), Dimensional Yale-Brown Obsessive Compulsive Scale (DY-BOCS) = 55 (18), and Global Assessment of Functioning (GAF) = 40 (19).

His medication was changed by titrating clomipramine up to 125 mg and haloperidol up to 4.5 mg/day. Thus, we assisted with a rapid resolution of the psychomotor arrest, with greater accessibility for the patient to dialogue, remission of the delusional idea of guilt, toward which the patient showed progressively more insight and experienced it more as an obsessive doubt. No rituals or compulsions were highlighted. The mood remained depressed, even though the facial expressions were progressively more relaxed and he spent less time in bed. Despite the improvement, C.M. always felt he was getting worse, and he did not believe that he could overcome the current episode. Depressive symptoms are mainly characterized by anhedonia, hypersomnia, difficulty concentrating, feelings of guilt, and inadequacy.

Cognitive function was investigated, but the results of the neuropsychological tests (global cognitive function, selective attention and attention-executive skills, language, memory, logical-deductive reasoning, frontal efficiency tests) appeared to be normal. Brain Magnetic Resonance Imaging (MRI) did not show significant abnormalities. From a diagnostic point of view, the Structured Clinical Interview for DSM-5 Clinician Version (SCID-5-CV) (20) was administered, showing that the criteria for the diagnosis of Schizoaffective Disorder and Obsessive-Compulsive Disorder were met. Furthermore, the Structured Clinical Interview for DSM-5 Personality Disorders (SCID-5-PD) (12) highlights the coexistence of avoidant, obsessive-compulsive and paranoid personality disorders. These data will be confirmed, as we shall see, by a subsequent evaluation, once the acute aspects have been resolved.

Despite the initial improvement, after 3 days there was a clinical flare-up. The patient appeared more confused, at times disorganized in behavior, again dominated by issues of guilt and unworthiness. Thoughts appeared accelerated, with elements of derailment, accompanied by motor restlessness and insomnia, effectively configuring a mood episode with mixed features. C.M. appeared distressed, with exacerbation of the delusional ideas of reference and persecution toward the other inpatients. Therapy was first modified by reducing clomipramine to 50 mg/day. The thoughts and behavioral disorganization were however worsening, configuring a picture of severe psychotic anxiety, with emergent suicidal ideation. Finally, it was decided to discontinue clomipramine and continue only with haloperidol therapy up to 6 mg. After a few days, we witnessed the remission of the most evident psychotic symptoms, with the persistence of persecutory ideas and guilt, lived at the time in an ego-syntonic and at times in an ego-dystonic way. C.M. was gradually less anxious, less restless, and less dominated by intrusive thoughts.

Once full remission of psychotic symptoms was achieved, we introduced a new antidepressant with a more sedative effect, which was trimipramine up to 80 mg/day. Agitation therefore reduced and, after 33 days of hospitalization, the patient was progressively less distressed and less gloomy. The mood seemed to improve both subjectively and objectively, in the absence of delusional ideation. The obsessive thoughts appeared less pervasive (although the interpersonal sensitivity and fleeting ideas of reference remained), the patient appeared to be less guarded with the medical staff. He was then discharged from our ward with a diagnosis of Major Depressive Episodes with Psychotic Behavior and Obsessive-Compulsive Disorder. We however suggested that the patient be admitted in a rehabilitation ward, in order to consolidate the achieved results. The patient accepted the suggestion. At the time of discharge, the scores of the rating scales showed significant improvement: BPRS = 29, HAM-D = 10, HAM-A = 8, YMRS = 5, PANSS = 44, DY-BOCS = 38, GAF = 65.

The patient follow up continued on an outpatient basis by psychiatric services, showing good compliance with the therapy and no further acute episodes at the one-year follow-up. Once the psychopathological compensation was reached and the acuity resolved, the patient was again subjected to personality assessment tests. The SCID-PD confirmed the diagnosis of avoidant, obsessive-compulsive, and paranoid personality disorder (20). The patient was also subjected to further psychometric scales, such as the short version of the Temperament and Character Inventory (TCI-125) (21), Magical Ideation Scale (MIS) (22, 23), Perceptual Aberration Scale (PAS) (24), and Hypomanic personality scale (HPS) (25).

As for the TCI-125 assessment, the results of the SCID-PD were in fact confirmed. The most significant traits appeared to be those relating to the Harm Avoidance dimension, with a score of 20, significantly higher than the average for age and sex (mean 8.05 ± 4.186). The scores of Novelty Seeking (2, mean 7.91 ± 3.244), Persistence (1, mean 3.21 ± 1.526), Self-Directeness (9, mean 18.92 ± 4.413), and Self- Transcendence (0, mean 5.96 ± 3.786) were instead lower than the average. Finally, the Cooperativeness score (18, mean 18.28 ± 3.470) and the Reward Dependence score (10, mean 7.68 ± 2.758) were slightly increased (21).

Evaluation with MIS demonstrated the presence of magical thinking, obtaining a score of 9/30, which appears higher than the Italian average for age and sex (mean 5.1 ± 4), testifying schizotypical traits (22, 23). The PAS showed a score of 13/35, which places the patient in a slightly above average position (mean 7.8 ± 5.77) (24), highlighting the ease in incurring erroneous and abnormal perceptions of reality. The HPS evaluation instead showed a result clearly below average, with a score of 8/48 (mean 21.08 ± 8.19) (25).

The re-evaluation of the patient at 1 year of follow-up also made it possible to evaluate the subjective experience of illness. The patient has good insight and good compliance with the proposed treatments, both pharmacological and psychological and rehabilitative, in the phases of clinical compensation. He appears to be very aware of his disorder, up to the point of identifying himself with the figure of “the patient,” who according to him allows him shelter from the world. Regarding his last hospitalization, C.M. shows a strong experience of shame, so he asks not to talk about it.

## Discussion

Personality development is a complex phenomenon that is affected by multiple genetic and environmental factors (26–30). The traditional delineation of Kretschmer's Sensitive Delusion of Reference emphasizes as a core element, the development of persecutory delusions driven by a specific personality type, usually triggered by a key experience where the feeling of shame is activated, often facilitated by a particular socio-cultural context (31). In this perspective, Kretschmer is rightly credited to have proposed one of the first theories of the psychogenic formation of delusions, which makes them easy to comprehend. What is usually less emphasized is the fact that in his landmark work *Der sensitive Beziehungswhan* (1918). Kretschmer goes at length in describing different courses of the Sensitive Delusion of Reference and in addressing the issue of the delimitation of this clinical presentation with others (1).

Regarding the course of the disorder, Kretschmer remarked that next to brief, self-limiting delusional episodes, which nowadays would fit the diagnostic criteria of acute and transient psychoses (32), some patients suffered from ongoing symptoms and difficulties for many years; a famous example of this chronic course of illness is that of Helene Renner, whose illness course Kretschmer described in detail.

Concerning the boundaries of the Sensitive Delusion of Reference with other disorders, Kretschmer stated that despite the fact that the disorder is sharply characterized, that does not mean that it is sharply delimited (1); in fact it may show overlaps with many other syndromes. In this regard, interestingly, the existence of an obsessive neurosis-like type presentation is explicitly mentioned and the possibility of a combination of the Sensitive Delusion of reference and “circular delusion” (corresponding to the current Bipolar Disorder) was hypothesized by Kretschmer.

An interesting aspect to consider is the phenomenologically oriented distinction that has been proposed between persecution (“Poor me”) paranoia and punishment (“Bad me”) paranoia (33). Whereas the first form of paranoia is more characteristerized by non-affective psychotic disorders, the second type is instead more frequent in non-clinical populations, as well as in cases of anxious-depressive experiences (34). Although in this clinical case it was possible to find both forms of paranoia at different times of the clinical course, the aspects of “Bad me” paranoia appeared to be predominant, confirming the presence of a mood disorder. The presence of punishment paranoia was also related to low self-esteem, negative self-evaluative thinking, and a tendency to develop negative evaluations about others, all factors involved in this case (33, 35).

Thus, the present case report is in our opinion a good example of how the Sensitive Delusion of Reference, as described by Kretschmer, may show interesting intersections with mood disorders. C.M. has always shown a prevalently depressive polarity, with experiences of inadequacy and marked interpersonal sensitivity also conveyed by his personality characteristics. However, it is possible to highlight in his history some characteristics of oscillation and cyclicality. In fact, while the major episodes with psychotic characteristics show environmental triggers, other mood alterations do not show such a clear correlation with life events. The case of C.M. clearly shows how the overlap between the sensitive personality and mood disorder gives rise to mixed pictures, which are difficult to categorize according to the DSM-5 nosography (6). In fact, in the intercritical phases, the aspects of interpersonal sensitivity and delusional interpretation prevail and then turn into experiences of guilt in the depressive phases and into experiences of persecution in the activated phases (1, 11, 36). In this case, the use of clomipramine seems to activate the patient, generating a picture of anxiety, restlessness, and disorganization, which went into remission only after the antidepressant therapy was stopped and the antipsychotic therapy increased. With regard to obsessive symptoms on the other hand, the fluctuating adhesion to the delusion of reference in the coexistence of anancastic personality characteristics as also confirmed by the SCID-PD, is expressed in characteristics similar to an Obsessive-Compulsive Disorder. The mood-dependent component of obsessive manifestations would make us think more of obsessive symptoms secondary to mood alteration (37, 38).

## Conclusion

This case report encourages a renewed interest in the concept of Kretschmer's Sensitive Delusion of Reference, open to the possibility that its typical manifestations may be linked not only to the sensitive personality but also to a mood disorder episode in progress. In the persistence of the background of interpersonal sensitivity and ideation of reference, one could then distinguish activated episodes, characterized by mood-incongruent persecutory delusion, and episodes of depression, dominated instead by ideation of guilt. Greater attention to the overlap between sensitive personality and affective disorders is obviously necessary to confirm this clinical observation.

## Data Availability Statement

The original contributions presented in the study are included in the article/supplementary material, further inquiries can be directed to the corresponding author/s.

## Ethics Statement

Written informed consent was obtained from the individual(s) for the publication of any potentially identifiable images or data included in this article.

## Author Contributions

All authors listed have made a substantial, direct and intellectual contribution to the work, and approved it for publication.

## Conflict of Interest

The authors declare that the research was conducted in the absence of any commercial or financial relationships that could be construed as a potential conflict of interest.

## Publisher's Note

All claims expressed in this article are solely those of the authors and do not necessarily represent those of their affiliated organizations, or those of the publisher, the editors and the reviewers. Any product that may be evaluated in this article, or claim that may be made by its manufacturer, is not guaranteed or endorsed by the publisher.
